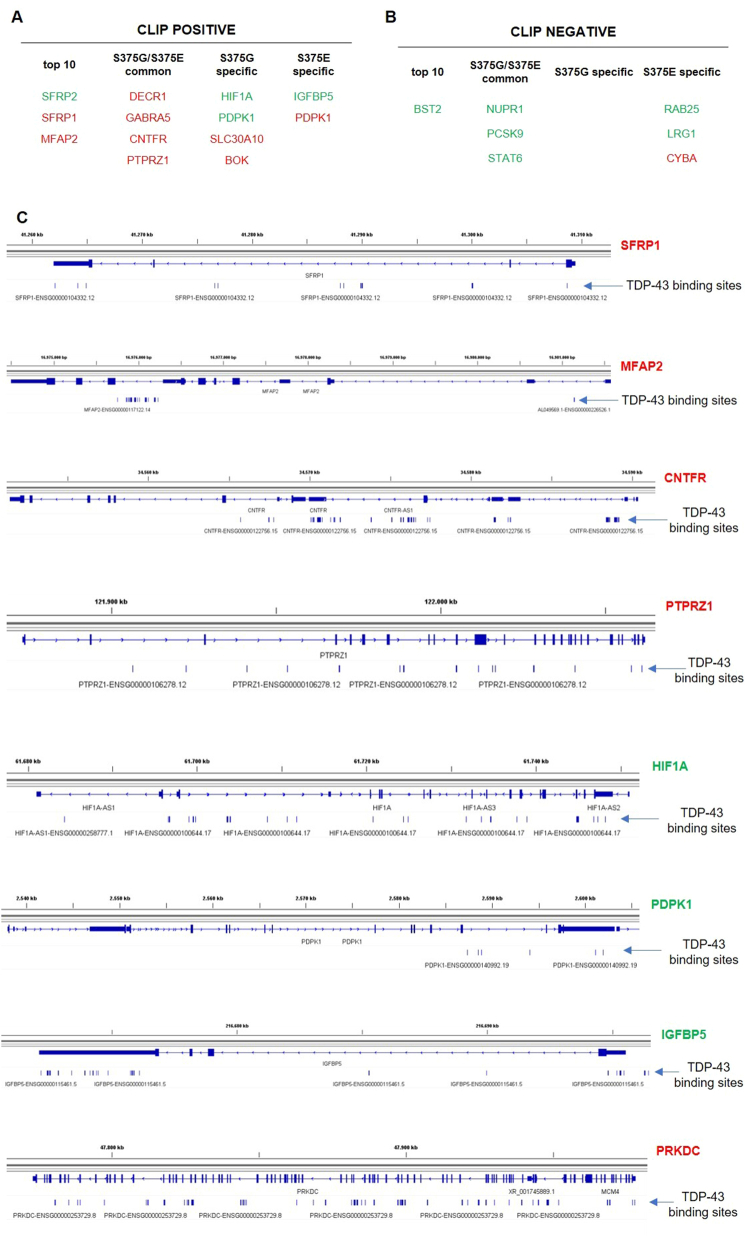# Unraveling the toxic effects mediated by the neurodegenerative disease–associated S375G mutation of TDP-43 and its S375E phosphomimetic variant

**DOI:** 10.1016/j.jbc.2022.102252

**Published:** 2022-07-12

**Authors:** Francesca Paron, Simone Barattucci, Sara Cappelli, Maurizio Romano, Christian Berlingieri, Cristiana Stuani, Douglas Laurents, Miguel Mompeán, Emanuele Buratti

**Affiliations:** 1 Molecular Pathology, International Centre for Genetic and Engineering Biotechnology (ICGEB), Trieste, Italy; 2 Department of Life Sciences, University of Trieste, Trieste, Italy; 3 “Rocasolano” Institute for Physical Chemistry, Spanish National Research Council, Madrid, Spain

**Keywords:** TDP-43, disease-associated mutants, phosphorylation, cell cycle, apoptosis, mitochondria, AIF1, AIF1, apoptosis-inducing factor 1, BSA, bovine serum albumin, CDK6, cyclin-dependent kinase 6, CLIP, crosslinking immunoprecipitation, CTD, C-terminal domain, DEG, differentially expressed gene, DMEM, Dulbecco’s modified Eagle’s medium, FC, fold change, GO, Gene Ontology, HEK293, human embryonic kidney 293 cell line, PTM, post-translational modification, TDP-43, TAR DNA-binding protein 43

## Abstract

TAR DNA-binding protein 43 (TDP-43) is a nucleic acid–binding protein found in the nucleus that accumulates in the cytoplasm under pathological conditions, leading to proteinopathies, such as frontotemporal dementia and ALS. An emerging area of TDP-43 research is represented by the study of its post-translational modifications, the way they are connected to disease-associated mutations, and what this means for pathological processes. Recently, we described a novel mutation in TDP-43 in an early onset ALS case that was affecting a potential phosphorylation site in position 375 (S375G). A preliminary characterization showed that both the S375G mutation and its phosphomimetic variant, S375E, displayed altered nuclear–cytoplasmic distribution and cellular toxicity. To better investigate these effects, here we established cell lines expressing inducible WT, S375G, and S375E TDP-43 variants. Interestingly, we found that these mutants do not seem to affect well-studied aspects of TDP-43, such as RNA splicing or autoregulation, or protein conformation, dynamics, or aggregation, although they do display dysmorphic nuclear shape and cell cycle alterations. In addition, RNA-Seq analysis of these cell lines showed that although the disease-associated S375G mutation and its phosphomimetic S375E variant regulate distinct sets of genes, they have a common target in mitochondrial apoptotic genes. Taken together, our data strongly support the growing evidence that alterations in TDP-43 post-translational modifications can play a potentially important role in disease pathogenesis and provide a further link between TDP-43 pathology and mitochondrial health.

Human TAR DNA-binding protein 43 (TDP-43) was isolated and described for the first time in 1995 as a transcriptional inactivator of integrated human immunodeficiency virus through its binding to the TAR DNA sequence (1). Subsequently, in 2001, TDP-43 was identified as a RNA splicing factor involved in the occurrence of a monosymptomatic form of cystic fibrosis (2). A few years later, in 2006, TDP-43 was discovered for the first time as the major ubiquitinated component of inclusion bodies in ALS and frontotemporal lobar degeneration patients' brains. Under pathological conditions, the protein is depleted from the nucleus and sequestered as hyperphosphorylated and ubiquitinated insoluble aggregates, disturbing the physiological nuclear functions of TDP-43 and its trafficking to the cytosol (3, 4). In recent times, considering the increasing number of diseases that are characterized by TDP-43 misregulation, a new term, “TDP-43 proteinopathies,” has been coined (5).

From a structural and functional point of view, TDP-43 belongs to the highly conserved heterogeneous nuclear ribonucleoprotein family (6). All heterogeneous nuclear ribonucleoproteins play an important role in multiple steps of gene expression regulation, including transcription, splicing, mRNA stability, DNA replication/repair, protein translation, and export or retention of nascent RNA (7) and, over the past few years, several members of this protein family have been found to be key players in neurodegeneration, especially within the ALS–frontotemporal degeneration disease spectrum (8, 9, 10, 11).

TDP-43 has been shown to be involved in multiple levels of RNA processing, such as splicing, transcription, transport, and translation. Under physiological conditions, TDP-43 is able to modulate its own protein levels through a negative feedback loop by binding its own mRNA in the 3′UTR region, leading to mRNA instability and degradation (12).

In TDP-43 proteinopathies, the protein is often aggregating in the cellular cytoplasm (although nuclear aggregates can also occur) and undergoes different post-translational modifications (PTMs), such as ubiquitination, phosphorylation, acetylation, sumoylation, and cleavage, to yield C-terminal fragments (13). Several studies performed on PTMs have highlighted their ability to regulate the interaction profile of TDP-43 with RNA and protein substrates and act as an important quality control checkpoint (14). By contrast, the N-terminal domain and C-terminal domain (CTD) of TDP-43 promote physiological oligomerization and association with stress granules and other condensates (15, 16, 17, 18). In general, these associations are reversible, and their conversion into pathological aggregates is prevented by PTMs. Nevertheless, cellular stress or other insults can upset this delicate balance, tipping the scale toward pathological TDP-43 aggregates (19, 20).

In parallel to PTMs, since 2008, many articles have reported disease-associated mutations in the *TARDBP* gene, supporting the idea of direct involvement of TDP-43 in neurodegenerative disorders, like ALS and frontotemporal lobar degeneration. In general, mutations in this gene are rare events (3% in familial ALS and 1.5% in sporadic ALS), but their characterization is important to better understand their functional significance and therefore TDP-43 pathogenic mechanisms (21). Interestingly, mutations can display both loss-of-function and gain-of-function characteristics (22). Loss-of-function mechanisms occur when mutations enhance the aggregation of TDP-43 or reduce the ability of the protein to bind to RNA. On the other hand, gain-of-function effects occur when mutations induce abnormal interaction with other protein factors or cellular components, thus disrupting important pathways for the maintenance of neuronal survival. Interestingly, most TDP-43 pathogenetic mutations lie in the CTD suggesting that they may affect the protein–protein interaction network or the post-translational status of the protein, leading to the development of TDP-43 proteinopathies (23). Very recently, it has been shown that disease-associated mutations in the C-terminal region can affect the condensation properties of TDP-43 by altering a conserved α-helical structure within this sequence and can selectively control the protein’s engagement to various RNA substrates (24).

Previously, we studied a particularly early onset of ALS case in a 26-year-old woman where DNA analysis of the *TARDBP* gene identified a S375G change, predicted to affect PTM with the elimination of a phosphorylation site (25). This variant was listed as “low frequency” in general patient sequencing databases. Therefore, to determine whether this substitution could be pathogenic, we performed preliminary functional assays, and it was observed that both the expression of S375G and of its “phosphomimic” variant (S375E) in HeLa cell line displayed alterations in the nuclear–cytoplasmic distribution (25). In this study, we sought to further investigate the reasons for this toxicity and better clarify the importance of TDP-43 PTMs in ALS as they might represent a potential future target for therapeutic options (26, 27, 28).

## Results

### Structural and functional analysis of the S375G and S375E stably expressing cellular clones

**Figure 1:**
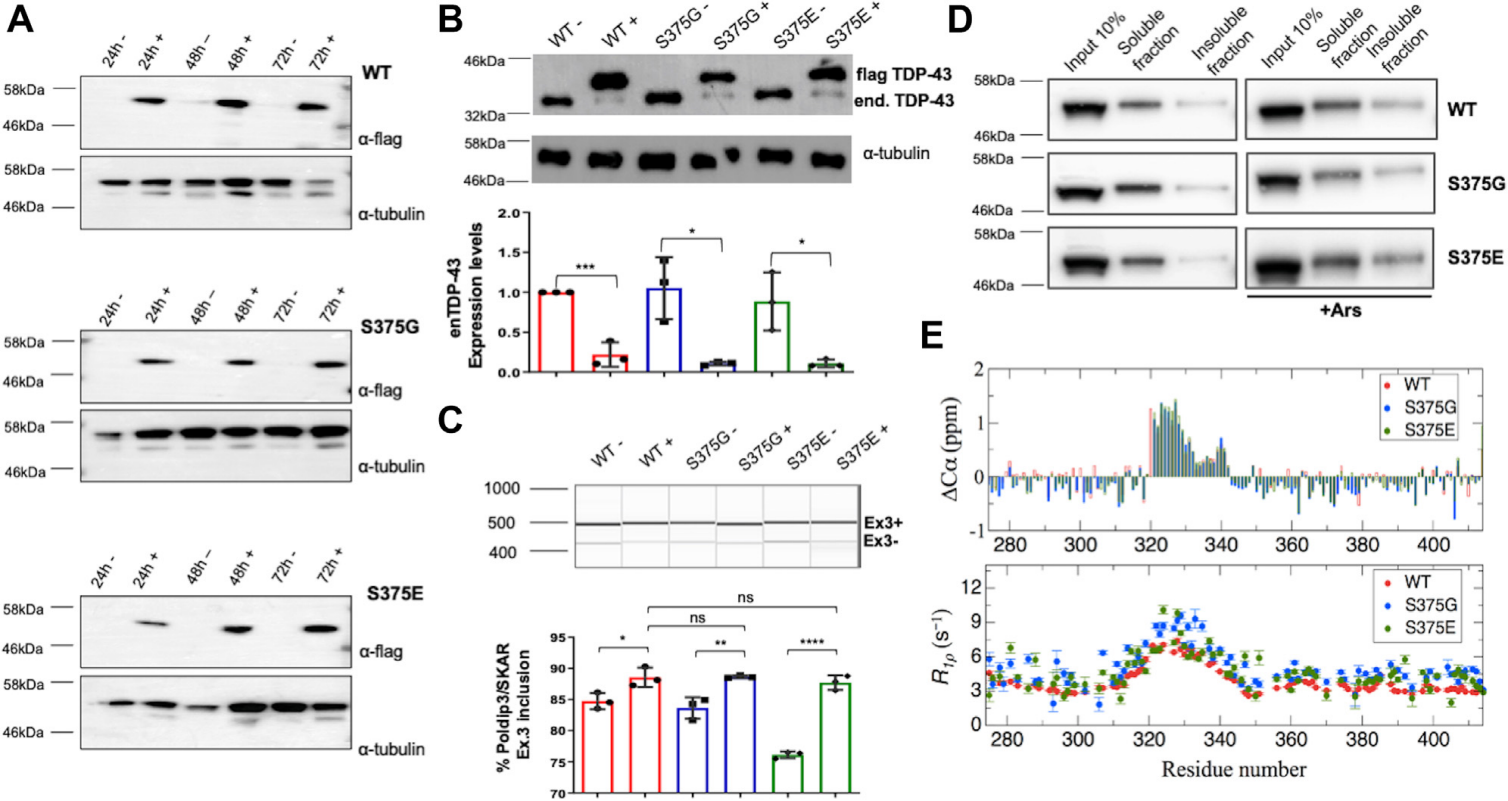
**Molecular analysis of TDP-43 WT, S375G, and S375E stably expressing cells.***A*, Western blot analysis of the three TDP-43 stable clones’ expression upon tetracycline induction at three different time points: 24, 48, and 72 h. The WT, S375G, and S375E proteins are specifically detected with α-FLAG antibody. Tubulin is used as a standard loading control. *B*, stable clones endogenous TDP-43 autoregulation at 48 h. In the *upper part* of the panel, Western blot analysis was performed against total TDP-43 detecting both the stably expressed protein and the endogenous TDP-43. Tubulin was used as a loading control. Western blot quantification of endogenous TDP-43 expression is plotted in a column graph (*red* for WT, *blue* for S375G, and *green* for S375E samples). The statistical analysis of endogenous TDP-43 expression levels was performed with multiple comparison one-way ANOVA test with Bonferroni’s correction using GraphPad software (GraphPad Software, Inc) and is reported in the *lower panel*. *C*, endogenous *POLDIP3* pre-mRNA splicing analysis in stably expressing cells was carried out with Qiaxcel capillary electrophoresis (*upper panel*). In this experiment, POLDIP3 exon 3 inclusion is plotted in a column graph (*red* for WT, *blue* for S375G, and *green* for S375E). Statistical analysis from three independent experiments was performed with multiple comparison one-way ANOVA test with Bonferroni’s correction using GraphPad software. *D*, solubility test on WT-, S375G-, and S375E-expressing clones. Western blot analysis was performed against total TDP-43 in endogenous conditions (*left*) as well as upon sodium arsenate treatment (*right*). *E*, conformational ^13^Calpha chemical shifts (*top panel*) for WT, S375G, and S375E TDP-43 CTD, calculated as ^13^Calpha (experimental) − ^13^Calpha (coil), where the latter are determined from the primary sequence using the parameters reported by Kjaergaard and Poulsen (103). Values higher than 0.3 ppm are indicative of partially populated alpha helix. Longitudinal relaxation rates (*bottom panel*) in the rotating frame for TDP-43 WT and S375 variants. CTD, C-terminal domain; TDP-43, TAR DNA-binding protein 43.

In order to identify the functional differences between the WT TDP-43 protein and the S375G TDP-43 mutant, we engineered stable mammalian cell lines (human embryonic kidney 293 [HEK293] Flp-In T-REx; Thermo Fisher Scientific) capable of expressing TDP-43 WT and S375G variants at similar levels upon addition of tetracycline. In addition, to better appreciate the possible functional consequences of phosphorylation at the S375 position, we also created a cell line expressing the S375E phosphomimetic mutant. We screened all stable clones to identify three cell lines that were expressing comparable levels of each TDP-43 protein at 24/48/72 h (Fig. 1*A*), and these clones were then used in all further experiments.

As a first functional analysis, we considered autoregulation of TDP-43 because it is well known that when this protein is in large excess it is able to bind a specific region in the 3′UTR of its own pre-mRNA, which induces skipping of intron 7 containing the main polyadenylation site (pA1), and triggers mRNA degradation (29). Therefore, we evaluated if expression of S375G and the S375E mutants might alter TDP-43 autoregulation compared with WT protein, as already described to occur for other disease-associated TDP-43 mutants, such as Q331K (30). As shown in Figure 1*B*, however, no statistical differences were observed in the autoregulation activity of S375G and S375E TDP-43 variants compared with WT protein. All three exogenous proteins, in fact, were able to downregulate the expression of the endogenous protein to the same low levels. In terms of looking for functional differences between mutants, this result is important because it suggests that any changes observed in the cells expressing the variants cannot be ascribed to differential residual levels of endogenous TDP-43 among clones.

**Supplementary Figure 1:**
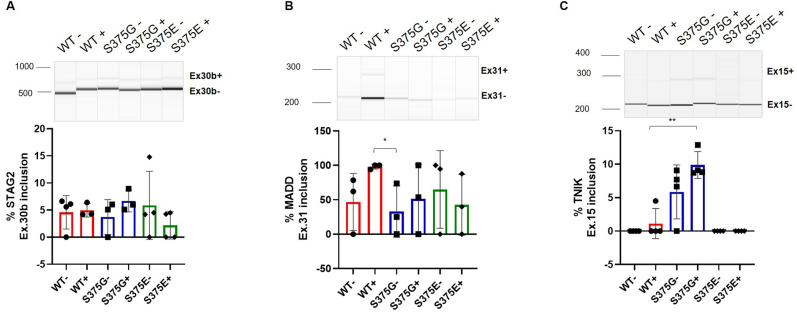


As a second functional analysis, we then compared the levels of endogenous POLDIP3 exon 3 inclusion in all three stable clones after 48 h upon the induction. The event was chosen because it represents one of the best readouts of TDP-43 splicing activity (31, 32). Figure 1*C* shows that following induction, both the S375G and S375E mutants behaved exactly like the WT protein, in terms of keeping the same inclusion/exclusion ratio of exon 3 following their induction (*i.e.*, 80% POLDIP3 exon 3 inclusion). These results fully support the conclusions we obtained with the CFTR exon 9 minigene add-back assay previously employed in transient transfection experiments using these mutants (25). The observed significant increase in exon inclusion between the minus and plus induction conditions can be explained by the fact that the expressed proteins have slightly higher expression levels than the endogenous TDP-43. In Fig. S1, we analyzed other well-known TDP-43 splicing targets, such as STAG2 exon 30b (Fig. S1*A*), MADD exon 31 (Fig. S1*B*), and TNIK exon 15 (Fig. S1*C*) (33, 34). Also, for these targets, the S375G- and S375E-expressing cells are behaving like the WT, except for TNIK exon 15 inclusion, in which the S375G stably expressing clone significantly increased exon recognition (Fig. S1*C*).

Subsequently, we evaluated if the S375G and S375E mutants might alter the solubility/insolubility of TDP-43 both in basal and under stress conditions following sodium arsenate treatment as previously described (35). However, Figure 1*D* shows that the soluble and insoluble fractions of both mutants were comparable to those from WT TDP-43 both in basal (Fig. 1*D*, *upper panels*) and under stress conditions (Fig. 1*D*, *lower panels*). These results suggest that neither mutation can affect the solubility properties of these TDP-43 variants compared with WT.

Finally, we also investigated whether the S375G and S375E mutants could alter some of the structural features of the C-terminal region (CTD) of TDP-43. Although this region is normally considered to be intrinsically disordered, residues 320 to 340 preferentially populate α-helical conformations (36), and other segments tend to adopt β-strands (37). Regarding a recently reported medium-resolution cryo-EM structure of the entire CTD in an amyloid-like conformation, S375 adopts a critical position in the middle of a central beta strand, where it abuts S393 and lies near S395 (38). All three of these serines are phosphorylated in *ex vivo* patient brains (39) but do not form part of the amyloid isolated from *ex vivo* patient brains (40). Thus, it is possible that these S375 mutations could alter conformational tendencies and might affect TDP-43 protein–protein interaction network, as highlighted in a recent work (24). Remarkably, the NMR chemical shift data and longitudinal relaxation measurements in the rotating frame (R_1rho_) reveal that these mutations alter neither the conformational tendencies (Fig. 1*E*) nor the dynamics of the soluble CTD as the profiles are nearly identical (*central panel*) as the averaged values over the 50 residues around the mutated sites (S375G: 4.34 ± 0.23 s^−1^; S375E: 4.01 ± 0.30 s^−1^; and WT: 3.24 ± 0.20 s^−1^) of soluble species of TDP-43 detectable by liquid-state NMR.

**Supplementary Figure 2:**
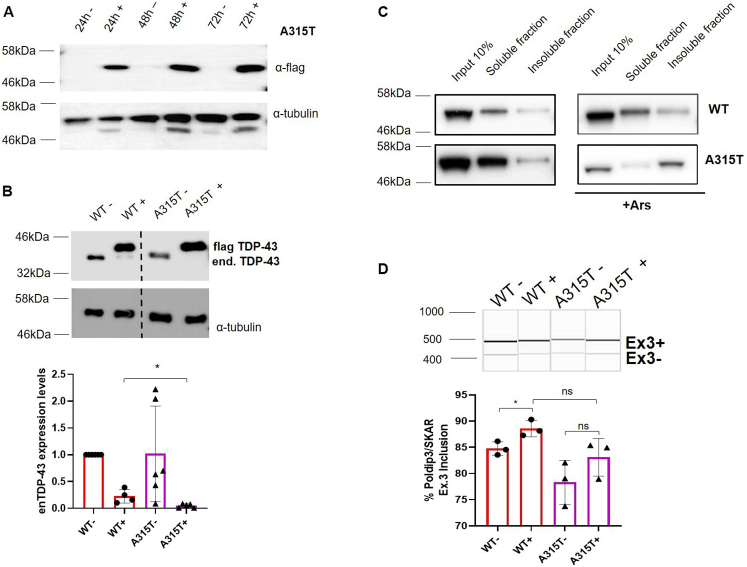


Considering the negative nature of these results, we then wanted to confirm that our assays were sensitive enough to detect changes and how these mutants compared with other more studied TDP-43 mutations. To address this issue, we used a HEK293 cell line expressing one of the classic and most studied disease-associated mutation: A315T. We used the A315T cell line to repeat all the biochemical experiments carried out with the S375G/S375E mutants in Figure 1. As shown in Fig. S2, similar levels of A315T expression compared with the S375G/S375E cell lines (Fig. S2*A*) showed specific changes in autoregulation (Fig. S2*B*) and solubility (especially following arsenite treatment; Fig. S2*C*). No changes were also observed with regard to POLDIP3 exon 3 splicing (Fig. S2*D*). Taken together, we conclude that our assays are sensitive enough to detect functional differences of the S375G/S375E mutants in our assays.

### Intrinsic fluorescence and light scattering measurements of TDP-43 CTD WT, S375G, and S375E

**Figure 2:**
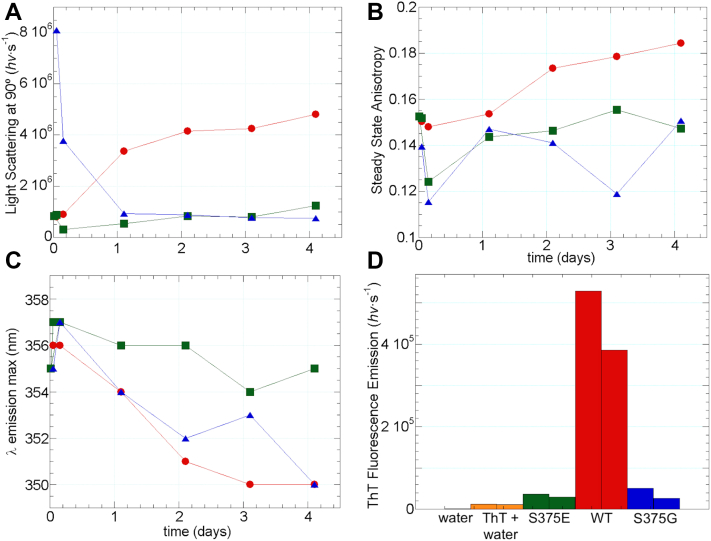
**Fluorescence measurements evince decreased self-association for the S375G and S375E variants.** Time courses of (*A*) 90° light scattering at 280 nm, (*B*) steady-state Trp (λexcitation = 280 nm, λemission = 375 nm), and (*C*) λ of maximal Trp emission for 30 μM WT (*red circles*), S375E (*green squares*), and S375G (*blue triangles*) in 20 mM Mes buffer, pH 6.7, 25.0 °C. *D*, ThT (25 μM) fluorescence (λexcitation = 440 nm, λemission = 480 nm) measured in the presence of water (*orange bars*), S375E (*green*), WT (*red*), and S375G (*blue*). The *lines* connecting the points do not represent a physical property but are meant as an aid to guide the eye. The *lower light* scattering, smaller Trp anisotropy, less *blue*-shifted Trp emission, and modest ThT fluorescence enhancement of the S375E and S375G variants reflect a reduced aggregation tendency.

To explore the aggregation potential, the intrinsic fluorescence (from Trp residues) and the 90º light scattering of the WT and S375G and S375E variants were evaluated from measurements made at different times. Unlike liquid-state NMR, these spectroscopic assays are sensitive to and can report on the behavior of large oligomers. The samples were matched to have the same concentration (30 μM) and were assayed in 20 mM Mes at pH 6.7. As shown in Figure 2, *A* and *B*, the WT sample scattered more light and showed a high Trp anisotropy than the S375G and S375E variants, which can be interpreted to mean that it has a stronger tendency to form aggregates. In addition, the Trp intrinsic fluorescence maximum is significantly *blue* shifted relative to S375E and modestly compared with S375G, indicating that the WT Trps are in a more hydrophobic milieu (Fig. 2*C*). Finally, ThT to a final concentration of 25 μM was added to sample aliquots, and spectra were recorded using the following parameters: T = 25 °C, scan speed = 2 nm s^−1^, λ_excitation_ = 440 nm, λ_emission_ = 430 to 520 nm, and bandwidths (excitation and emission = 2 nm). Whereas S375E and S375G show modest ThT fluorescence enhancements relative to ThT in water, the increase for WT is over 10-fold higher (Fig. 2*D*). This is indicative of the formation of amyloid-like structures. Based on these observations, we infer that the S375G and S375E seem less prone to *in vitro* self-association and aggregation than the WT protein in these *in vitro* experiments. The effect of the S375E phosphomimetic variant is in line with recent results (41) reporting that phosphorylation solubilizes TDP-43. The increased solubility of the S375G variant can be rationalized by considering that Gly is a very small and flexible residue that strongly destabilizes β-sheet structure (42), including β-strands of amyloid structures.

### Changes in cell appearance in S375G and S375E variants

**Figure 3:**
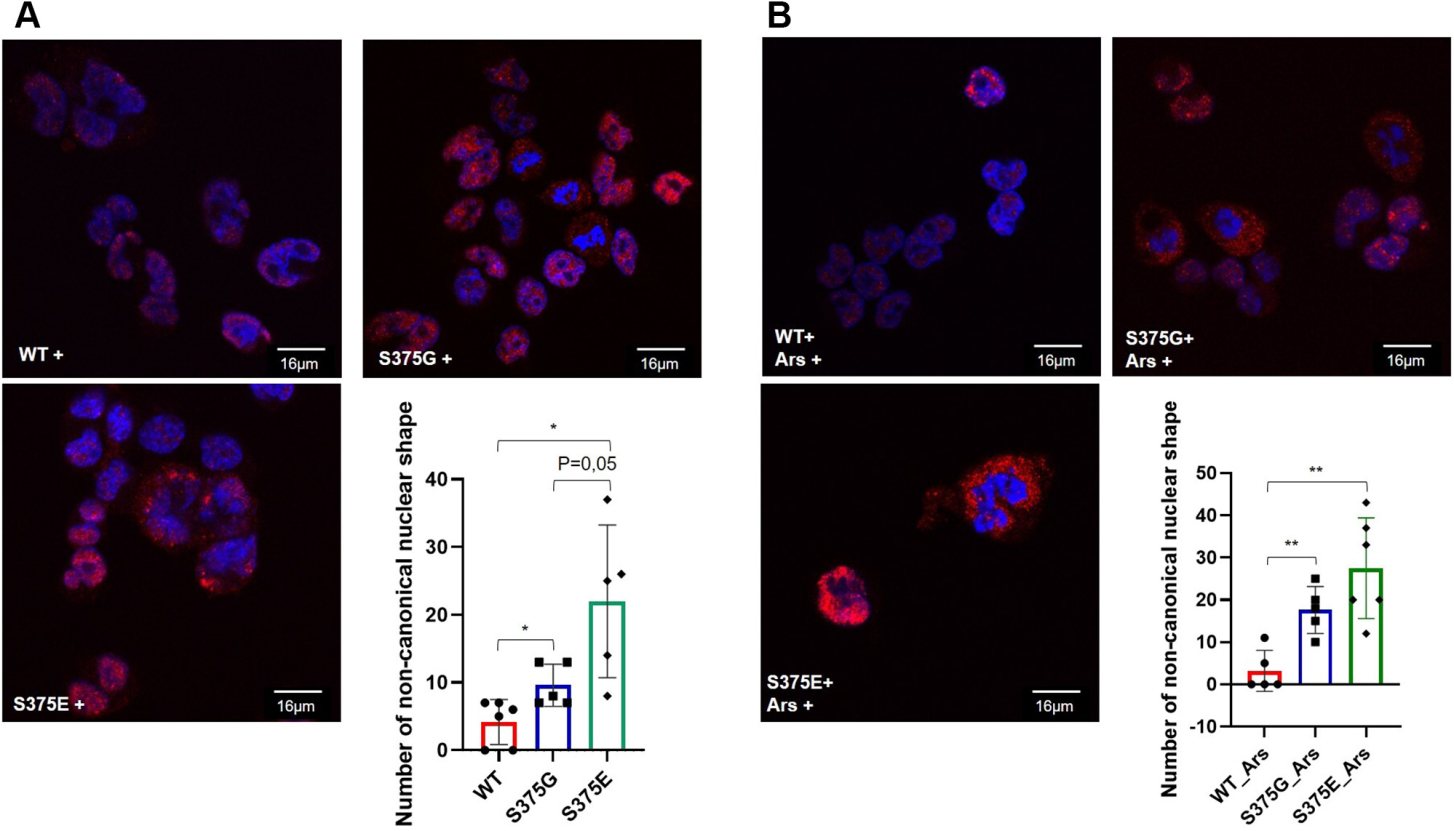
**Immunolocalization analysis of TDP-43 WT-, S375G-, and S375E-expressing clones.***A*, the following merged immunolocalization plots were reported for each clone where the nuclei (DAPI channel) are stained in *blue*, whereas the Alexa Fluor 594 *red channel* detects α-FLAG TDP-43 expression. The count of nuclei with noncanonical shape is reported in a column graph (*red* for WT samples, *blue* for S375G, and *green* for S375E). Statistical analysis was performed with multiple comparison one-way ANOVA test with Bonferroni’s correction using GraphPad software (GraphPad Software, Inc). *B*, the same analysis was also repeated for each clone following sodium arsenate treatment. DAPI, 4′,6-diamidino-2-phenylindole; TDP-43, TAR DNA-binding protein 43.

**Supplementary Figure 3:**
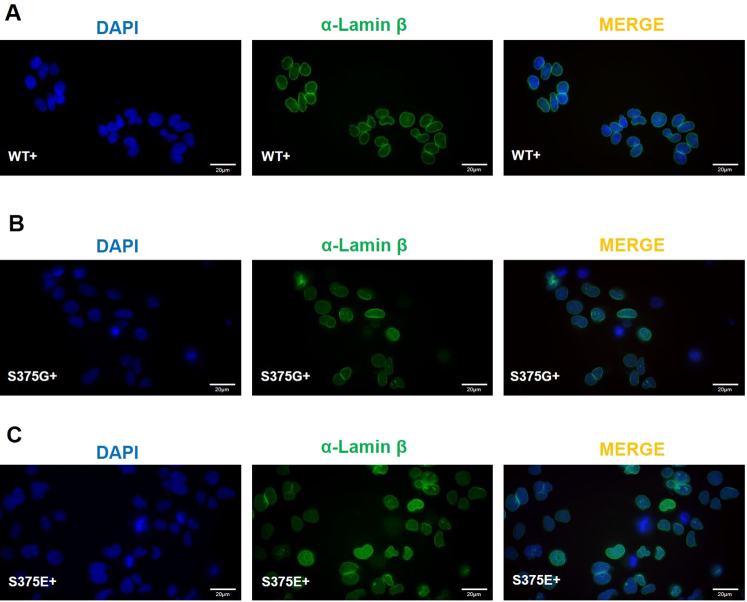


Immunolocalization analyses of the stably expressing mutants revealed that in both the S375G and S375E stable clones, there was an abundance of nuclei with a noncanonical shape, showing altered appearance (Fig. 3*A*). Importantly, in basal conditions, this finding was particularly evident for the phosphomimic S375E clone that showed 19.67% of cells with nuclear shape changes, compared with the S375G-expressing clone (9.60%) and the WT clone (6.25%). This observation was even more striking under stress conditions (Fig. 3*B*). In fact, following sodium arsenite treatment, the number of cells with nuclear-shape alterations increased in both the mutant isoforms (S375E = 27.50% and S375G = 20%) compared with the WT clone (9.33%). Supporting the idea of a loss in the normal nuclear conformation, the WT-, S375G-, and S375E-expressing cells were stained for lamin β, an important constituent of the nuclear lamina. As reported in Fig. S3, the lamin β signal seems to be more diffuse or less consistent in S375G and S375E cells that are showing the abnormal nuclear phenotype. This phenomenon is not occurring in WT cells.

Changes in nuclear shape have recently been associated with control in cell cycle with nuclear flattening being recently reported to be required for G1 to S transition in HeLa cells (43) as well as transcriptional profiles (44) by affecting the amount of chromatin brought in proximity to the nuclear lamina.

**Figure 4:**
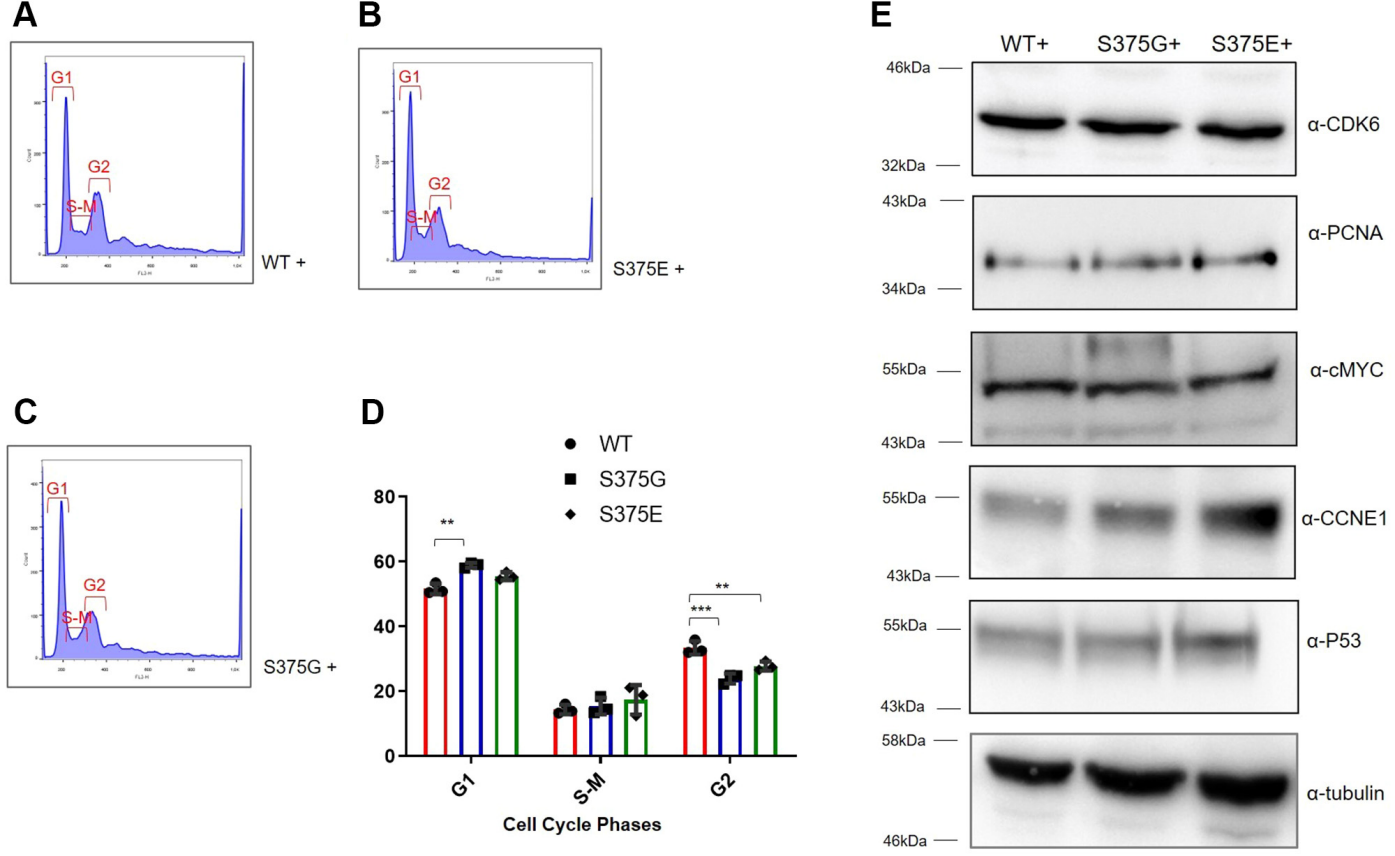
**Cell cycle analysis on the TDP-43 WT, S375G, and S375E expressing clones.***A*, propidium iodide flow cytometry analysis on the TDP-43 WT-expressing cells: G1, S–M, and G2 phases are reported. *B*, propidium iodide flow cytometry analysis on the TDP-43 S375G-expressing cells: G1, S–M, and G2 phases are reported. *C*, propidium iodide flow cytometry analysis on the TDP-43 S375E-expressing cells: G1, S–M, and G2 phases are reported. *D*, the number of cells in each cell cycle phase from three independent experiments is plotted in a grouped graph (*red* for WT samples, *blue* for S375G, and *green* for S375E). A multiple comparison one-way ANOVA test was performed using GraphPad software (GraphPad Software, Inc). *E*, Western blot analysis against some gene involved in the cell cycle (α-CDK6, α-PCNA, α-cMYC, α-CCNE1, and α-P53). Tubulin was used as a standard loading control. TDP-43, TAR DNA-binding protein 43.

**Supplementary Figure 4:**
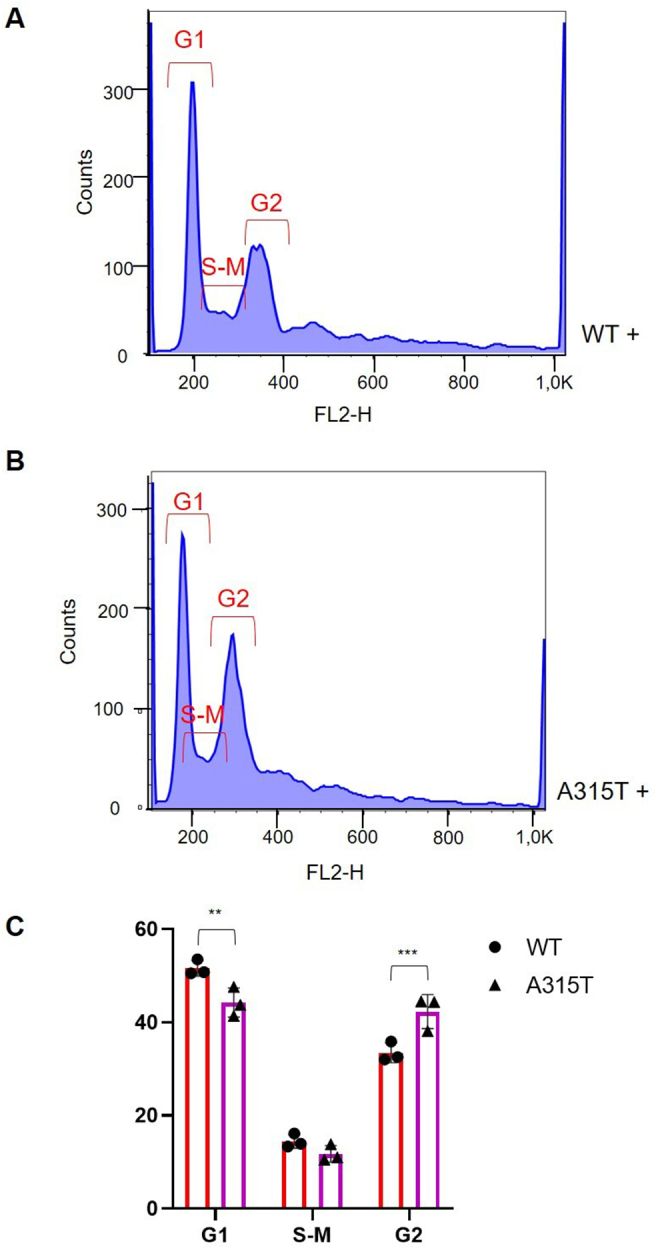


Therefore, to verify if these morphological observations were correlated with alterations of cell division, the proportion of cells in each phase of the cell cycle was determined by flow cytometry (Fig. 4, *A*–*C*). This analysis showed that the percentage of S375G and S375E cells in G2 phase was significantly lower than that of WT cells. Moreover, the percentage of S375G cells in G1 phase was significantly higher in comparison to those of WT cells (Fig. 4*D*). Interestingly, the same cell cycle analysis performed on the stably expressing A315T cells (Fig. S4*B*) showed an opposite direction to the one observed in the S375G and S375E cell lines with an increase in G2 phase and decrease in G1 phase (Fig. S4*C*).

These results suggested that an accelerated progression of cycling cells through the cell cycle (rather than decreased percentage of cells in G0 phase) might be responsible for higher rates of proliferation in S375E and S375G cells. Considering the known TDP-43–dependent regulation of cyclin-dependent kinase 6 (CDK6) expression that was shown to be accompanied with nuclear shape changes (45), we therefore tested if overexpression of S375E and S375G was associated with variations in CDK6 protein levels. However, no differences in CDK6 expression were observed in both TDP-43 mutant clones compared with WT (Fig. 4*E*). In addition to CDK6, we tested also other proteins known to be involved in cell cycle regulation: the proliferating cell nuclear antigen (PCNA), which plays a role during DNA replication; the MYC proto-oncogene (cMYC), involved in the cell cycle progression; cyclin E1 (CCNE1), which works in complex with other members of the same family in order to achieve the transition between the G1 phase and S phase; and tumor protein P53 (P53), known for its role in cancer and cell cycle arrest. However, no significative changes were reported for the TDP-43 S375G- and S375E-expressing clones in comparison to the WT.

### RNA-Seq analysis of the stable clones supports an involvement of apoptotic and cell proliferation pathways by S375 phosphorylation

**Figure 5:**
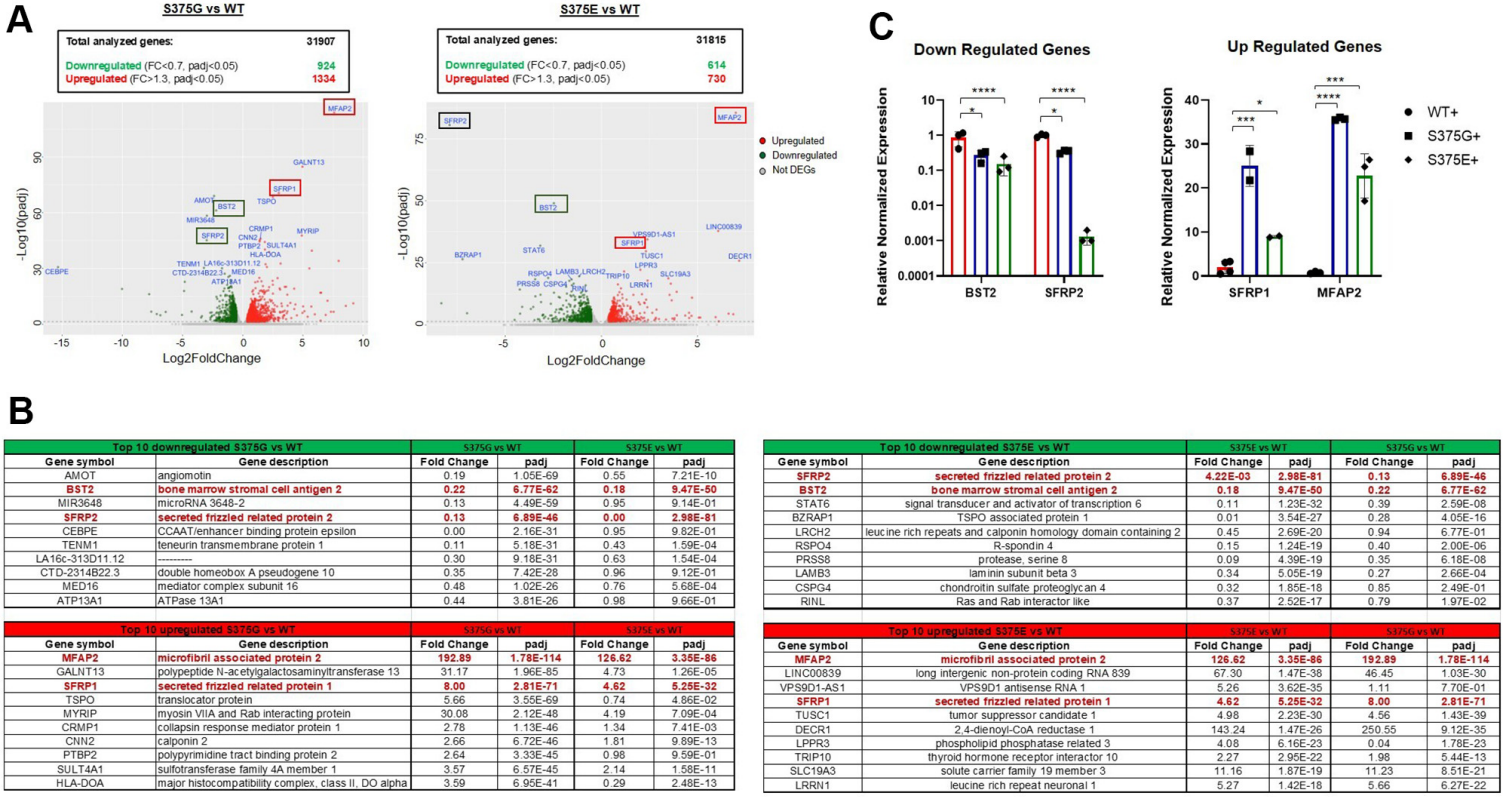
**RNA-Seq analysis of S375G and S375E *versus* WT stable clones.***A*, volcano plot representation of differentially expressed genes (DEGs) of S375G and S375E TDP-43 mutants with respect to WT TDP-43 (control). Upregulated and downregulated genes are reported in *red* and *green dots*, respectively. Not DEGs are also showed as *gray dots*. *Top* 10 DEGs are indicated in *blue*, and commonly regulated genes among S375G and S375E are highlighted with *colored boxes* (*red* for upregulation and *green* for downregulation). *B*, list of *top* 10 upregulated and downregulated genes for both S375G and S375E mutants (the corresponding values in each category are also indicated). Tables report the gene symbol, gene description, and corresponding fold change and padj values. *C*, RT–quantitative PCR (qPCR) RNA-Seq validation from three independent experiments of the *top common* DEGs between S375G- and S375E-expressing clones. In the grouped graph, WT, S375G, and S375E expression changes are reported in *red*, *blue*, and *green*, respectively. Statistical analysis was carried out by using an unpaired *t* test with the GraphPad software (GraphPad Software, Inc). TDP-43, TAR DNA-binding protein 43.

To further investigate the effects of these variants on transcription, RNA-Seq was applied to total mRNA extracts from the S375G and S375E stable cell lines to fully analyze their gene expression compared with WT-expressing cells. An overview of the differential expression analysis (cutoffs: padj < 0.05 and 0.7 < fold change [FC] > 1.3) performed on each mutation is reported in Figure 5*A*.

Regarding the S375G clones, the total number of differentially expressed genes (DEGs) was 2258 (out of the 31,907 analyzed genes), among which 924 were downregulated and 1334 were upregulated. On the other hand, after overexpression of the S375E mutation, the total number of DEGs was 1344 (out of 31,815 analyzed genes), among which 614 were downregulated and 730 were upregulated. For each condition, the name of selected DEGs is reported in the corresponding volcano plot (Fig. 5*A*), and the top upregulated and downregulated genes are listed in Figure 5*B*.

**Supplementary Figure 5:**
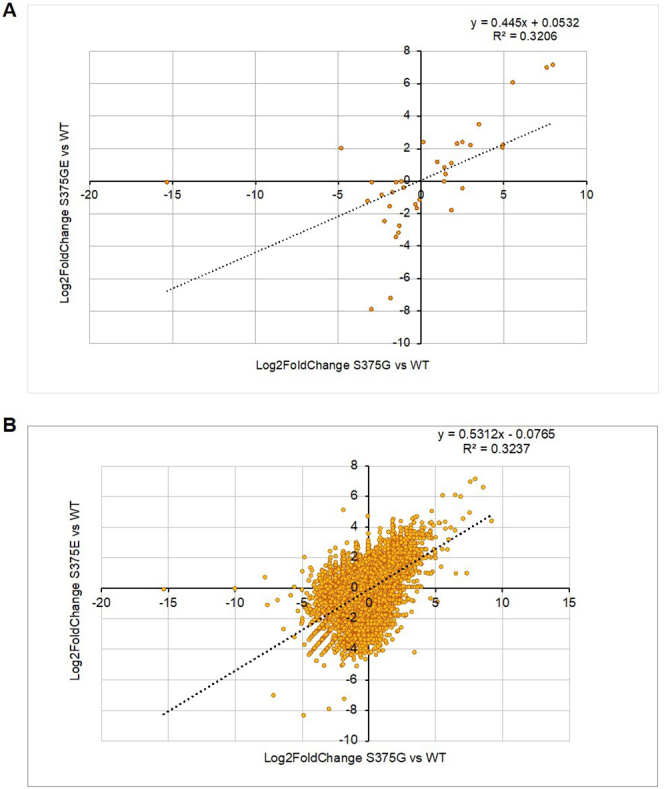


Interestingly, the overlap between the top downregulated and upregulated genes is not complete, and this observation suggests that the variations in gene expression induced by the S375G mutant do not follow the same pathways as that induced by the S375E mutation (further discussed later). As shown in Figure 5*B*, in fact, there are only four common DEGs in the S375G and S375E cell lines (*BST2*, *SFRP2*, *MFAP2*, and *SFRP1*) in the top 10 DEGs in both lines. Independent validation of their expression changes reported in Figure 5*C* shows that with the only exception of *BST2* gene, the relative FC of the other three genes *SFRP2*, *SFRP1*, and *MFAP2* is considerably different between the two variants. This difference is evident if we compare the corresponding value of every top 10 genes in each cell lines, where many of these genes are commonly altered in both cell lines but at widely different levels. To better appreciate the overall similarity of gene expression, we then plotted the log_2_ S375G FC *versus* the log_2_ S375E FC for both the top 10 genes and the whole genes analyzed in the study (Fig. S5, *A* and *B*, respectively). As shown in this figure, in both cases, the *R*-squared value was rather small, supporting the presence of a clear difference between these two mutants.

Nonetheless, among these four commonly regulated genes, three of four possess a direct link to apoptosis. In fact, in both cell lines, *SFRP2* is highly downregulated, and this gene has been shown to be protective against apoptosis (46, 47). Conversely, in both cell lines, the *SFRP1* gene is upregulated, and it has been shown that increased expression of this gene can increase apoptosis in human U251 glioma cells (48), in immortalized human osteoblasts (49), and it has been demonstrated that its strong upregulation can lead to a mitochondria-dependent apoptotic pathway, leading to cytochrome *c* release (50). Most importantly, of particular interest with regard to the apoptotic process is the downregulation in both cell lines of *BST2* (also known as CD317), as knockdown of this factor in serum-deprived tumor cells has been shown to impair mitochondria function and subsequently promote the release and nuclear translocation of apoptosis-inducing factor 1 (AIF1) (51).

### Increased AIF1 expression in the mutant-expressing cell lines

**Figure 6:**
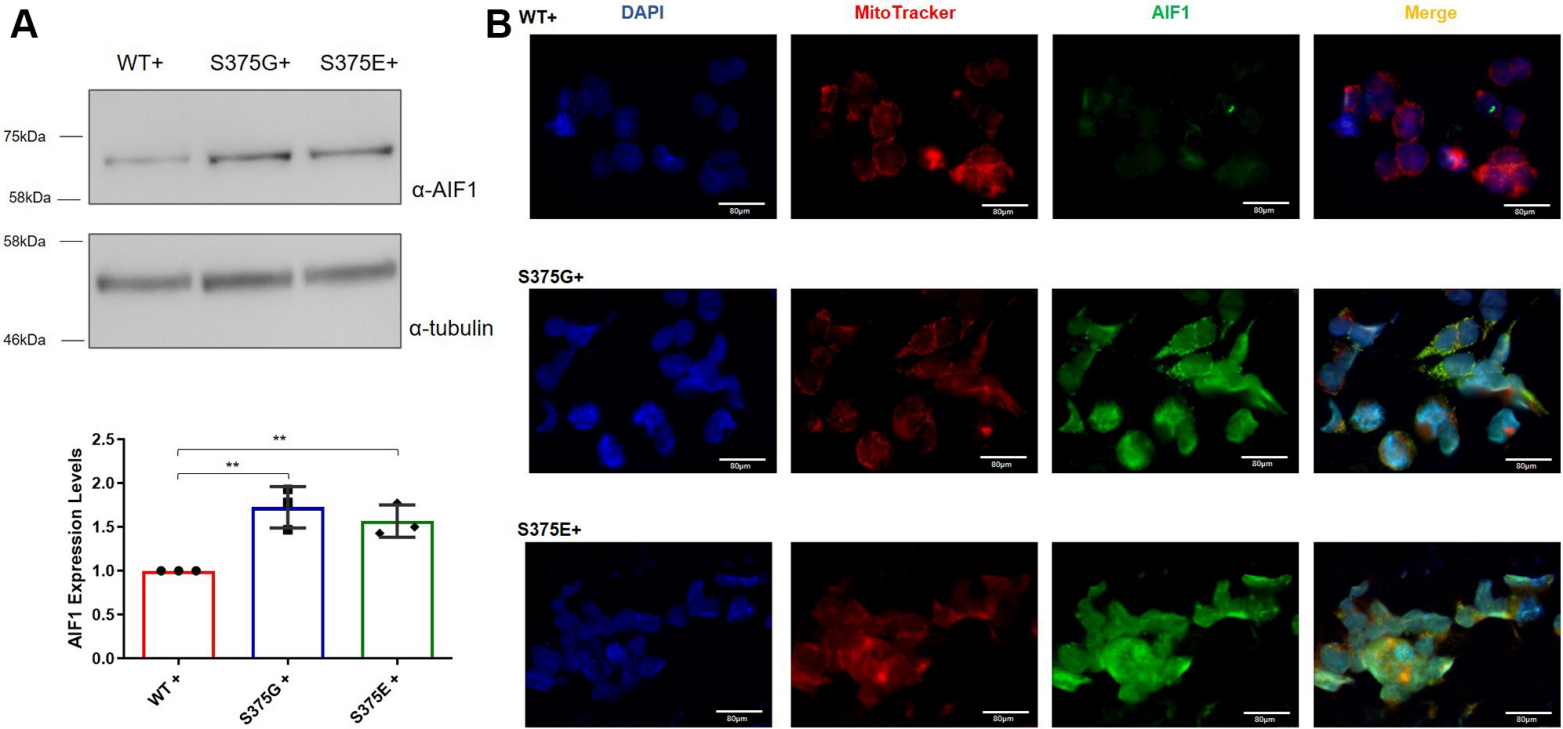
**AIF expression and immunolocalization analysis on the TDP-43 WT-, S375G-, and S375E-expressing cells.***A*, in the *upper panel* is reported a Western blot analysis of AIF1 expression with tubulin used as a standard loading control. The amount of expressed AIF in the WT- (*red*), S375G- (*blue*), and S375E- (*green*) expressing cells is plotted in a *column graph* from three independent experiments. Statistical analysis was performed with multiple comparison one-way ANOVA test with Bonferroni’s correction using GraphPad software (GraphPad Software, Inc; n = 4). *B*, immunolocalization analysis of AIF1 protein. The nuclei are stained *blue*, mitochondria in *red* from the MitoTracker *Red* CMXRos kit (Thermo Fisher Scientific); and the AIF1 signal is stained *green* with α-mouse Alexa Fluor 488. Merged channels are also reported. The scale represents 80 μm. AIF, apoptosis-inducing factor; TDP-43, TAR DNA-binding protein 43.

Based on this consideration, we then decided to check whether downregulation of *BST2* was followed by induction of AIF1 protein in our stable cell lines. Indeed, we found an increase in expression of the mitochondrial AIF1 that contributes to DNA fragmentation and chromosomal condensation in cells undergoing early apoptosis (52) and that has been described to be consistently involved in neuronal death (53, 54). In particular, following a lethal signal or a strong and persistent cellular stress, AIF1 protein translocates *via* cytosol to the nucleus where it binds to DNA and provokes caspase-independent chromatin condensation (55). As shown in Figure 6*A*, AIF1 expression was found to be increased in both S375G- and S375E-expressing clones. This observation was also consistent with the immunolocalization experiment where AIF1 signal intensity was more robust in the mutants compared with WT (Fig. 6*B*). Moreover, in addition to expression changes, AIF1 subcellular localization was also altered supporting the hypothesis of mitochondria-related apoptosis. As shown in Figure 6*B*, in the WT-expressing clone, the AIF1 (in *green*) was mainly localized with the mitochondria signal (*red*) as expected. Mitochondrial colocalization was also present for the S375G mutant although part of the protein remained mislocalized in the nucleus, and this result that was even more evident for the phosphomimic S375E mutant.

From a biological point of view, the significance of this finding could be very high because, although rescue of TDP-43 pathology did not occur in a yeast model of disease following ablation of the homologous yeast gene (Aif1p) (56), elevated levels of this factor have been observed in specific molecular subtypes of ALS patients (57), in the cortex of mice subjected to mild traumatic brain injury (58), and very recently in Atxn-CAG100 knock-in mice that eventually develop TDP-43 pathology (59).

### Additional evidence for mitochondrial alterations in the stably expressing cell lines

**Figure 7:**
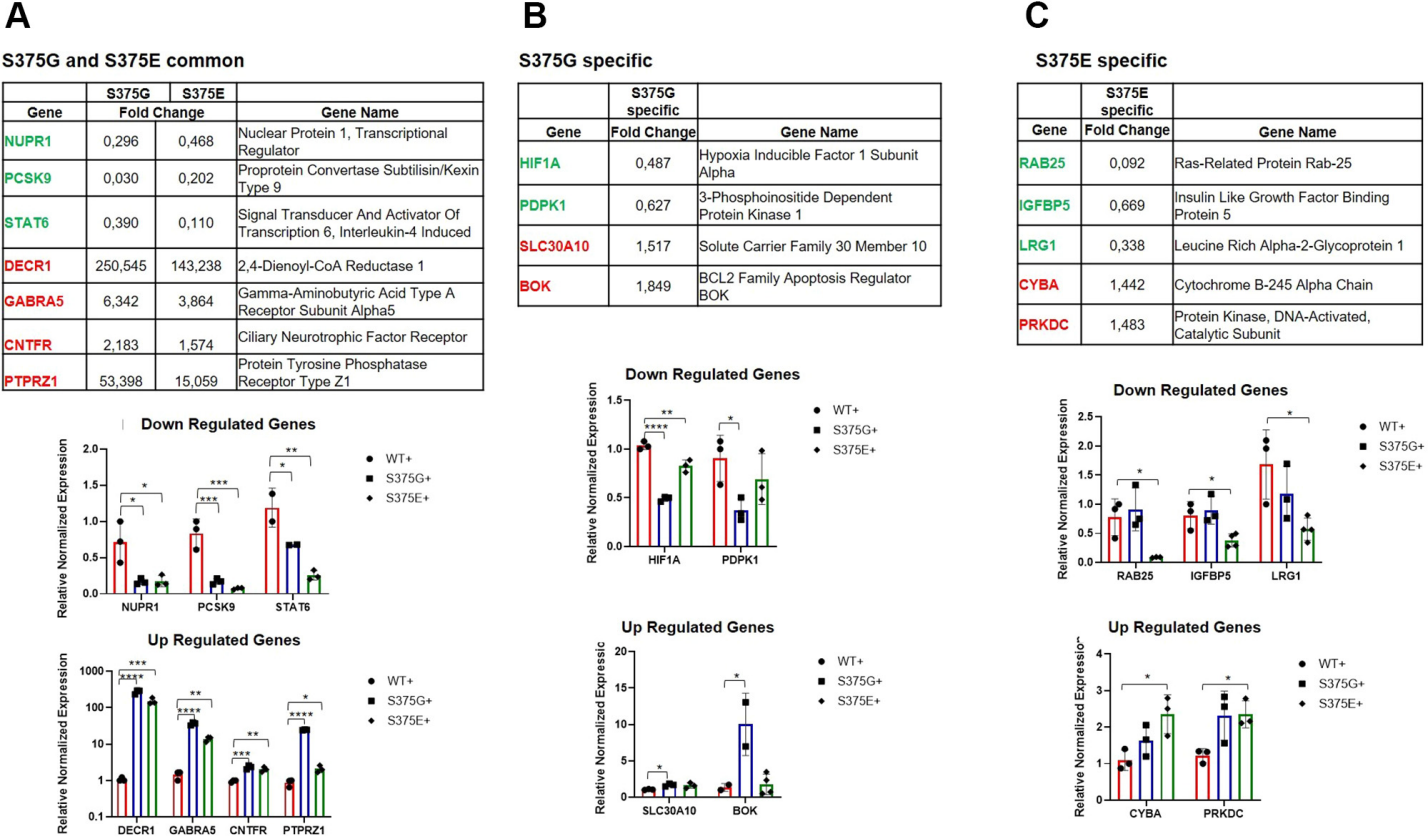
**RNA-Seq quantitative PCR (qPCR) validation analysis on gene related to neuronal/mitochondrial apoptosis.** In the *top halves* of each panel, a table with the gene names and their fold change is reported for differentially expressed genes (DEGs) in the neuronal/mitochondrial pathway that are (*A*) common in S375G- and S375E-expressing cells and those that are specific for (*B*) S375G-expressing cells and (*C*) S375E-expressing cells. Downregulated genes are highlighted in *green* and the upregulated genes in *red*. In the *lower part* of each panel, RT–qPCR validation performed on three independent experiments, plotted of these genes, is reported (WT, S375G, and S375E values are plotted in *red*, *blue*, and *green*, respectively). Statistical analysis was carried out using an unpaired *t* test with the GraphPad software (GraphPad Software, Inc).

Although the alteration of AIF1 was just related to the protein expression, additional support for the effect of these mutations on mitochondrial apoptosis has also emerged from the RNA-Seq analysis. For example, a common downregulated target between the S375G and the S375E clones is represented by the nuclear protein 1 transcriptional regulator (*NUPR1*), which induces loss of the mitochondrial membrane potential, inducing mitochondria-dependent oxidative stress (60) (Fig. 7*A*). These changes are also extended to other genes important for mitochondrial apoptosis that include *protein convertase subtilisin/kexin type 9* (*PCSK9*) and *signal transducer and activator of transcription 6* (*STAT6*) that are downregulated similarly to *NUPR1* as well as *2,4-dienoyl-CoA reductase 1* (*DECR1*), *gamma-aminobutyric acid type A receptor subunit alpha5* (*GABRA5*), *CNTFR*, and *PTPZR1* that are commonly upregulated (Fig. 7*A*).

Importantly, downregulation of the *PCSK9* gene has already been linked to the release of mitochondrial proapoptotic factors. This gene encodes for the neural apoptosis–regulated convertase 1, and its downregulation (through siRNA knockdown) showed an increase of the Bax/Bcl-2 ratio, the release of the cytochrome *c* from the mitochondria, and the activation of the caspase 3/caspase 9 cascade (61, 62). Likewise, the downregulation of the *STAT6*, *interleukin-4 induced*, a transcription factor related to interleukin-4 immune response, has also been associated with upregulation of apoptosis by affecting the ratio between Bax/Bcl-2 proteins (63) and RNAKL expression (64).

Regarding upregulated factors, *DECR1* is already known to play a role in fatty acid metabolism and oxidation in the mitochondrial compartment (65), whereas the *GABRA5* encodes for the α5 subunit of the receptor for gamma-aminobutyric acid, the major inhibitory neurotransmitter of the mammalian brain. Interestingly, *GABRA5* overexpression was described in different brain areas of Alzheimer's disease mouse model, and it was associated with a gamma-aminobutyric acid activation–dependent cell death, provoked by mitochondrial oxidative stress (66). Similarly, upregulation of the *ciliary neurotrophic factor receptor* (*CNFTR*) gene has been related to neuroprotective signaling (67). This factor was found increased in multiple sclerosis patients’ cortical neurons, as a potentially compensatory mechanism to the chronic cellular stress (68) and as a mediator of α-synuclein (SNCA)–induced neurotoxicity, *via* NF-κB signal transduction pathway and apoptotic proteins like Bcl-xl and Bax (69). Supporting this view, *SNCA* is also one of the upregulated genes that emerged from the RNA-Seq. Finally, from a neurodegeneration point of view, it is interesting to note that *PTPRZ1* has been identified as a potential schizophrenia susceptibility gene (70) where proteomic studies have shown that protein dysregulation events are mostly associated with mitochondrial function and oxidative stress responses (71).

On top of these common targets, the S375G and S375E mutant proteins regulate the expression of specific targets related to mitochondria-induced apoptosis (Fig. 7, *B* and *C*). For example, in the S375G-expressing clone, the *hypoxia-inducible factor 1 subunit alpha* (*HIF1A*) and the *3-phosphoinositide-dependent protein kinase 1* (*PDPK1*) are altered (Fig. 7*B*). All these genes have already been described to protect the mitochondrial membrane potential and to prevent mitochondria-related apoptosis in different cellular models, including neuronal cells (61, 72, 73). Moreover, the RNA-Seq analysis revealed upregulation of the *translocator protein* (*TSPO*), the *solute carrier family 30 member 10* (*SLC30A10*), and the *BCL2 family apoptosis regulator BOK* (*BOK*) (Fig. 7*B*). Like the downregulated factors, all these upregulated genes were described to enhance the mitochondrial membrane permeability and the release of proapoptotic factors (74, 75, 76).

Considering the S375E mutant, there are also several examples of upregulated or downregulated genes, specific for the mitochondrial pathway (Fig. 7*C*). In support of this proapoptotic effect, the downregulation of the *Ras-related protein RAB25*, of the *insulin-like growth factor–binding protein 5* (*IGFBP5*) and the *leucine-rich alpha-2-glycoprotein 1* (*LRG1*), has also been described to promote a mitochondria-dependent apoptosis in a variety of cells including neuronal ones (77, 78). Finally, the upregulation of the *cytochrome B-245 alpha chain* (*CYBA*) and *protein kinase, DNA-activated, catalytic subunit* (*PRKDC*) add additional evidence to the impairment of mitochondrial function and to the release of cytochrome *c* and other proapoptotic proteins in the S375E cell lines (79, 80, 81).

### S375G and S375E clones differentially affect cellular processes

**Supplementary Figure 6:**
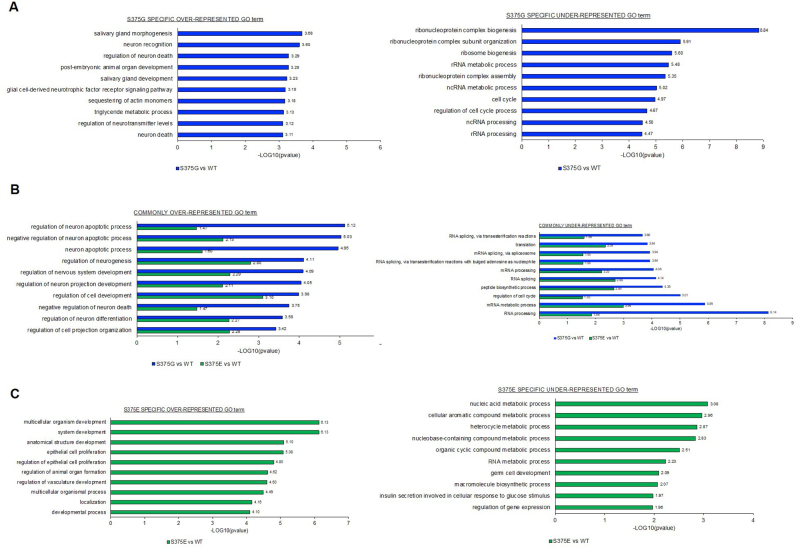


Interestingly, however, when scrutinizing at the global changes in gene expression between the two cell lines, it is also clear that both mutants do not have completely overlapping gene categories (Fig. S6). From this figure, it is evident that over-represented Gene Ontology (GO) terms for the S375G mutant compared with WT are predominantly connected with neuronal function regulation (Fig. S6*A*), whereas for the S375E phosphomimic, the most represented GO terms compared with WT are linked to cell proliferation and development (Fig. S6*C*). Importantly, among the common GO terms, the most important category is neuronal apoptosis (Fig. S6*B*). Interestingly, there are also significant differences in the under-represented GO terms for both S375G and S375E, with RNA metabolism under-represented for S375G and compound metabolic processes for S375E. In keeping with our previous results, a common under-represented GO term in both mutants is RNA splicing and other processes (Fig. S6*B*).

Finally, to add some mechanistic evidence of the detected changes and the affected cellular processes, we performed a search based on a TDP-43 crosslinking immunoprecipitation (CLIP) analysis performed by Tollervey *et al.* (82). As a result, Fig. S7 reports all CLIP-positive (Fig. S7*A*) and all CLIP-negative targets (Fig. S7*B*) of our validated genes. Among the CLIP-positive targets, we selected the best representatives in terms of TDP-43-binding sites (Fig. S7*C*). As shown in this figure, many validated genes are enriched in TDP-43-binding sites that are present in the middle of the pre-mRNA sequence as well as in the 3′UTR regions. Considering TDP-43 capability to modulate splicing, mRNA stability, RNA transport, and more in general RNA metabolism, the observed changes could therefore be related to a downstream effect promoted by the two TDP-43 variants in the way TDP-43 molecules cooperate.

Taken together, these results strongly suggest that the natural phosphorylation of the S375 residue in TDP-43 might considerably affect the properties of this protein even in normal conditions, thus highlighting the importance of PTMs in their ability to affect normal TDP-43 functions.

## Discussion

In ALS, TDP-43 mutations are quite rare events, and it is not always easy to prove a clear relationship between the discovered variant and disease (21). Nonetheless, many of them seem to affect potential phosphorylation sites in several ways: by removing a potential site of phosphorylation, by inserting a serine or a threonine (thus creating a novel hypothetical phosphorylated residue), or by missense substitutions that introduce negatively charged amino acid residues such as glutamic acid or aspartic acid, thus mimicking a phosphorylation event (21, 23). All these alterations can change the protein–protein interaction profile of TDP-43, increase its aggregation, affect half-life of the protein, and its nuclear–cytoplasmic localization. The type of phosphorylation can also be used to differentiate TDP-43 aggregates in distinct pathological subtypes. Recently, in fact, it has also been shown that phosphorylation of serine 369 can specifically detect in immunohistochemistry type B and C TDP-43 pathological inclusions but not type A (83).

Several studies have contributed to identify phosphorylation-affecting mutations as potentially playing a key role between the physiological and pathological balance of TDP-43. For example, the N352S mutation was described to enhance TDP-43 aggregation and insolubility, and the G295S mutation was found to induce twisted amyloid-like fibers that increased TDP-43 aggregation propensity (84, 85). This was also confirmed by the description of other mutations such as R361S and N390S that have been shown to increase the production of CTD truncated forms (85) that are often associated with promoting aggregation. Furthermore, in 2009, Corrado *et al.* (86) described a novel mutation (S396L) with a deleterious effect on the protein structure that was leading to the formation of low–molecular weight fragments of approximately 32 kDa. Finally, Ki Yoon Kim *et al.* (87) studied three different TDP-43 phosphomimic mutants (S379E, S403/404E, and S409/410E) and showed that they were all able to reduce Drosha stability, preventing protein–protein interaction and compromising TDP-43 function, thus inducing neurotoxicity in Neuro 2A cell line. However, in very few cases, it was possible to establish the exact pathway through which these mutations can drive neurotoxicity.

In this work, we show that the neurotoxic effects of the S375G and S375E mutations could be principally related to a neurotoxic effect because of the protein overexpression of specific genes, such as *AIF1*, and the upregulation and downregulation of other genes involved in the mitochondrial apoptotic pathway. Many recent observations have linked the presence of mitochondrial problems in several neuronal populations in the presence of TDP-43 aggregation or toxic C-terminal fragment expression (88, 89, 90, 91, 92). Moreover, TDP-43 itself has been described to be present in mitochondrial fractions of transgenic mice overexpressing the Q331K disease-associated mutant (93) and interact with mitochondrial proteins to influence the dynamics of these organelles (94). Indeed, inhibition of TDP-43 mitochondrial localization has been shown to block its neuronal toxicity (95). Taken together, all these observations suggest that mitochondrial dysfunction could play an important role in TDP-43 proteinopathies, as recently reviewed in detail (96), and the results from our study add to this line of evidence. Another interesting aspect is that it might have been expected to observe opposite effects for the two opposite conditions: S375G (loss of phosphorylation) and S375E (constitutive phosphorylation). However, the fact that similar functional effects can be detected following opposite actions has already occurred for TDP-43. In fact, it has been recently described to occur for parkin, where it has been recently reported that in patient fibroblasts carrying PGRN mutations, both the silencing and overexpression of TDP-43 led to a decrease in parkin expression levels (97). A possible explanation for this apparently contradictory observation was that TDP-43 is probably part of a complex that affects parkin expression: if TDP-43 is absent, the complex does not work and parkin expression decreases. On the other hand, when TDP-43 is overexpressed, the stoichiometry of the complex is likewise disrupted, and this would also result in parkin depletion. In our case, we can propose that a similar situation might occur with the S375 phosphorylation event: where there might be the need for TDP-43 to be present in complexes containing a specific balance of phosphorylated/unphosphorylated S375 residues. Any forced changes in this status, such as the ones that would occur in the case of the S375G and S375E variants, could then lead to similar consequences because in both conditions the complex would not be able to function properly.

For this reason, further analyses will be required in the future to better characterize the involved pathways, the kinases eventually involved in S375 phosphorylation, and whether the genes identified in these pathways could also play a pathological role in the absence of disease-associated mutations. Nonetheless, the observation that a single phosphorylation event can induce such prominent changes in genes controlled by TDP-43 is an important observation also with regard to the normal function of this protein and supports the notion that even relatively small changes in the post-translational status of TDP-43 can have profound influences on cellular metabolism. Therefore, our data from these variants further support the view that PTMs of TDP-43 might act as powerful modulators in TDP-43 normal functioning and neurodegenerative processes (14, 98).

## Experimental procedures

### Generation of inducible cell lines expressing TDP-43 WT, A315T, S375G, and S375E

HEK293 Flp-In T-REx cells were maintained in Dulbecco’s modified Eagle’s medium (DMEM) + Glutamax, supplemented with 10% tetracycline-free fetal bovine serum (Life Technology), 1% antibiotic antimycotic solution (Sigma–Aldrich), and 15 μg/ml blasticidin S HCL (Thermo Fisher Scientific). For stable transfections, cells were cotransfected with a mixture of pOG44 plasmid and recombinant pcDNA5 FRT/TO vector carrying either the WT or mutant (A315T, S375G, and S375E) version of the human FLAG-tagged TDP-43 (TARDBP; NM_007375) open reading frame. For transfections, Effectene Transfection Reagent (Qiagen) was employed following the manufacturer’s instructions. After 24 h, cells were seeded in p100 plates to select monoclones with 100 μg/ml hygromycin (Thermo Fisher Scientific). Monoclonal colonies were expanded under double selection (blasticidin and hygromycin). All experiments using the stable clones were performed after 48 h from the induction of TDP-43 expression with 1 μg/ml tetracycline (Sigma–Aldrich).

### Sodium arsenite treatment

In order to induce cellular stress, the cells were treated with 0.5 mM sodium arsenite for 40 min, as previously described in detail (35). After media substitution, cells were collected or processed for the immunofluorescence analysis.

### Mitochondria marker: MitoTracker Red CMXRos

Mitochondria of living cells were stained with *red* fluorescent probes of the MitoTracker Red CMXRos kit (250 mM, 2 min; Thermo Fisher Scientific), according to the manufacturer's instructions. After media substitution, cells were prepared for the immunofluorescence assay.

### Propidium iodide cell cycle assay and flow cytometry analysis

Half a million HEK293 Flp-In T-REx cells were plated in p35 with the DMEM/tetracycline selective medium. After 48 h from the induction, cells were collected, resuspended in 300 μl of ice-cold PBS and 700 μl of 96% ethanol (Honeywell), and incubated at −20 °C for 2 h. Subsequently, cells were centrifuged 5 min at 500*g*, and the supernatant was discarded. The pellet was resuspended and incubated for 15 min at room temperature in the dark with 500 μl of 0.1% Nonidet P40 (Thermo Fisher Scientific) in PBS supplemented with RNase (Sigma–Aldrich) (10 mg/ml), and propidium iodide (5 μg/ml) (Sigma–Aldrich). Flow cytometry was performed using FACS Calibur (Becton Dickinson). The gates were set based on physical parameters (side and forward scattered) and excluding/distinguishing the aggregates from the dividing cells (relation between area and breadth). The histogram representing the percentage of the events in the different phases of the cell cycle (G1, G2, and S/M) was then created by applying a third gate comparing the height and signal intensity. The raw data were analyzed using FlowJoVX software (FlowJo LLC, Becton Dickinson).

### Immunofluorescence assays

At day 1, cells were carefully washed once with 2 ml of PBS. Then, the cells were fixed with 2 ml of 3.2% paraformaldehyde (Electron Microscopy Science) in PBS for 1 h at room temperature. Subsequently, the slides were washed three times with 2 ml of PBS, and cell permeabilization was performed with 2 ml of 0.3% Triton (Sigma–Aldrich) in PBS (5 min on ice). After three washes with 2 ml of PBS, efficient quenching was obtained by incubating the specimens directly with PBS supplemented with 2% bovine serum albumin (BSA; Sigma–Aldrich) (20 min at room temperature). The slides were incubated upside down with 60 μl of the primary antibody diluted in 2% BSA/PBS solution in a dark humid chamber at 4 °C overnight. The day after, the slides were washed twice with 2 ml of PBS. The slides were incubated upside down with 60 μl of the secondary antibody diluted in 2% BSA/PBS solution for 1 h in a dark humid chamber. In the meantime, the SUPERFROST coverslips (Thermo Fisher Scientific) were cleaned with 100% isopropanol (Honeywell). Then, the slides were picked up with 1 ml of PBS, and they were washed twice with 2 ml of PBS. The slides were blocked face down on the coverslip with 18 μl of Vectashield with 4′,6-diamidino-2-phenylindole (Vector Laboratories, Inc). The slides were blocked definitively with nail polish and stored in dark at 4 °C. The slides were analyzed with Nikon Elements AR 4.40.00 64-BIT confocal microscope (Nikon) and LEICA epifluorescent microscope (Leica).

### Solubility assay: soluble–insoluble fractionation

Half a million HEK293 Flp-In T-REx cells were plated in p35 with the DMEM/tetracycline selective medium. After 48 h from the induction, cells were collected and resuspended in 1 ml of 10× radioimmunoprecipitation assay buffer (Cell Signaling Technology). After 2 min at room temperature, samples were mixed by rotation for 30 min at 4 °C. Subsequently, they were centrifuged at 7000*g* for 30 min at room temperature. Supernatants were transferred to 1.5 ml tube and disrupted by sonication using BioRuptor UCD-200 (Diagenode) for 5 min at high impulse (30 s on, 30 s off). Protein quantitation was achieved using Bradford assay (Bio-Rad). At this point, 60 μg of sonicated samples were mixed with 10 μl of 4× NuPAGE LDS Sample Buffer (Thermo Fisher Scientific), representing the input. On the other hand, 600 μg of lysate were mixed with 10× radioimmunoprecipitation assay buffer (Cell Signaling Technology) to achieve a final volume of 500 μl, were transferred to Beckman polycarbonate thick wall centrifuge tubes (Beckman Coulter), and ultracentrifuged with the Optima L-90K Ultracentrifuge (Beckman Coulter) at the following conditions: 121,968*g*, 1 h, and 25 °C. Then, each supernatant and pellet were mixed with 4× NuPAGE LDS Sample Buffer and loaded in a SDS-PAGE gel, following a denaturation step.

### Western blot assay

Western blot assays were performed with Power Blotter Semidry Transfer System (Thermo Fisher Scientific). To control the correct transfer, nitrocellulose membranes were stained with Pierce Reversible Protein Stain Kit for nitrocellulose membranes (Thermo Fisher Scientific), and the image was acquired with Alliance 9.7 Western Blot Imaging System (UVITEC). Membranes were blocked with 4% skimmed milk (nonfat dry milk) or 3% BSA in PBS and 0.1% or 0.01% Tween-20, depending on the antibody. The blocking step lasted for around 1 h. The primary antibody was incubated overnight at 4 °C and, the day after, three washes with PBS and 0.1% or 0.01% Tween-20 were performed (5 min each).

## Antibodies

The following antibodies were used in this study for Western blots and immunohistochemistry: α-FLAG M2 (Sigma–Aldrich), α-TDP-43 (Proteintech EU), α-AIF1 (Invitrogen), α-CDK6 (Santa Cruz Biotechnology), α-PCNA (Sigma–Aldrich), α-cMYC (Proteintech EU), CCNE1 (Santa Cruz Biotechnology), α-P53 (Cosmo Bio), and α-lamin β (Santa Cruz Biotechnology). The α-tubulin antibody was homemade and used as standard loading control. Secondary antibodies for Western blot analyses were α-rabbit horseradish peroxidase and α-mouse horseradish peroxidase Dako, whereas the α-rabbit and α-mouse Alexa Fluor 488 or 594 (Invitrogen) were used as the secondary antibodies for the immunofluorescence experiments.

### RNA extraction

RNA extraction was carried out by using the RNeasy Mini Kit (Qiagen) according to the manufacturer's instructions. The primer sequences used for amplification of POLDIP3 exon 3 inclusion levels are as follows: 5′-gcttaatgccagaccgggagttgga-3’ (forward) and 5′-tcatcttcatccaggtcatataaatt-3’ (reverse).

### RNA-Seq analysis of DEGs

Total RNA was extracted from WT (control), S375E, and S375G TDP-43 stable cell lines using miRNeasy Kit (Qiagen). Library construction and RNA-Seq were performed by Novogene (https://en.novogene.com/) on three independent clones obtained for each tested sample. RNA-Seq analysis was performed using Illumina HiSeq NovaSeq 600 instrument.

The original raw data from Illumina were transformed to sequenced reads by CASAVA base recognition. Low-quality reads (meaning reads with more than 50% nucleotides quality less than five or reads with more than 10% reads uncertain nucleotides) and reads containing adapters were removed from the analysis. Clean reads were mapped to the reference genome (GRCh37/hg19) using STAR software (version 2.5). Differential gene expression analysis was carried out using DEseq2 R package (version 2_1.6.3). The overall distribution of DEGs was evaluated using the following cutoff: for upregulated genes, FC >1.3 and padj <0.05; for downregulated genes, FC <0.7 and padj <0.05. Goseq package from R (version 3.14.3) (99) was also used for GO analysis of RNA-Seq data. For each mutation, significant over-represented and under-represented GO terms in the Biological Process category were considered (*p* < 0.05). The primers used for RT–quantitative PCR RNA-Seq validation are reported in Table S1.

### Structural analyses

Production of the TDP-43 C-terminal regions with the WT sequence and carrying the S375G and S375E mutants has been carried out as previously described (100). Briefly, CTD constructs with an N-terminal hexa histidine tag were expressed in *Escherichia coli* BL21 star cells in M9 media supplemented with ^15^NH_4_Cl and ^13^C-glucose and purified using nickel–nitrilotriacetic acid agarose beads that were washed with denaturing buffer (20 mM Tris–Cl, 500 mM NaCl, 10 mM imidazole, 1 mM DTT, 8 M urea, pH 8.0), with a final elution using an imidazole gradient (26). The purified polypeptides were applied into a PD-10 desalting column pre-equilibrated with 1 mM DAc to obtain a final concentration of 10 μM and at pH = 4.0. These conditions afford a direct comparison of the chemical shift and spin relaxation measurements following our recent characterization of the WT construct (101). NMR spectroscopy was performed using a Bruker Neo 800 MHz (^1^H) NMR spectrometer equipped with a cryoprobe and Z-gradients. The S375E and S375G variants' ^1^H–^15^N heteronuclear single quantum coherence spectra were assigned based on the WT spectrum and 3D HNCA, HNCO, and CBCA(CO)NH spectra. Conformational chemical shifts were used to evaluate possible alternations in backbone conformational tendencies in the variants. For determination of R_1rho_ rates, the CTD constructs were buffer-exchanged in 20 mM Mes (final pH 6.7 and final protein concentration adjusted to 30 μM), and eight hsqctretf3gpsi experiments with delays of 8, 60, 36, 300, 80, 200, 100, and 156 ms were recorded with a spin-lock RF field strength of 1.9 kHz. An exponential function was fit to the peaks’ intensities at different time delays to determine the R_1rho_ rates and their experimental uncertainties.

### Fluorescence and light scattering analysis of WT, S375G, and S375E variants

Fluorescence measurements were performed on a Jobin Yvon Fluoromax 4 spectrophotometer (HORIBA scientific) at 25 °C using 2 nm excitation and emission slit widths. The protein concentration was 30 μM for all three TDP-43 variants, and samples contained 20 mM deuterated acetic acid (pH = 6.7) and 0 mM KCl or 5 mM KCl. The excitation wavelength was calibrated using the most intense Xe emission line at 397 nm, and the water Raman signal was used to calibrate the emission wavelength. The scan speed was 2 nm s^−1^. Fluorescence spectra were recorded over 270 to 400 nm using an excitation wavelength of 280 nm. Light scattering at 90° was measured as the signal with both the excitation and emission wavelength set to 280 nm. The steady state fluorescence anisotropy was measured as (VV − VH)/(VV + 2VH), where VV is the signal measure with both excitation (at 280 nm) and emission (357 nm) polarizers set in vertical, and where VH is the signal measured with the excitation polarizer in vertical and the emission polarizer in horizontal (90°); anisotropy values of <0.10 and >0.20 are typical of mobile and rigid Trp indole moieties, respectively (102). After 4 days of measurements, aliquots were taken, and thioflavin T was added from a stock solution prepared in the same 20 mM Mes buffer, to a final ThT concentration of 25 μM. The ThT fluorescence spectra in the presence of water or 30 μM TDP-43 CTD WT, S375E, and S375G domains was measured at 25.0 °C using 2 nm slit widths, a 440 nm excitation wavelength, and scanning emission over 430 to 520 nm at 2 nm s^−1^. The same Jobin Yvon Fluoromax 4 spectrophotometer was used.

## Data availability

The dataset generated for this study have been deposited in the Gene Expression Omnibus of the National Center for Biotechnology Information and are accessible through Gene Expression Omnibus Series accession number GSE167385 (https://www.ncbi.nlm.nih.gov/geo/query/acc.cgi?acc=GSE167385).

## Supporting information

This article contains supporting information.

## Conflict of interest

The authors declare that they have no conflicts of interest with the contents of this article.

## Figures and Tables

**Supplementary Figure 7:**